# Artificial molecular motors induce a mechanical response in cells

**DOI:** 10.1038/s42004-021-00531-0

**Published:** 2021-06-17

**Authors:** Alberto Moscatelli

**Affiliations:** Communications Chemistry, https://www.nature.com/commschem

## Abstract

Mechanical forces can induce a biochemical response in cells. Now, it is shown that a molecular motor can exert enough force on the surface of a cell to induce a biochemical response too.

Cells can sense a mechanical perturbation and produce a biochemical response to help them adapting to the new environment. A particularly mechanosensitive part of a cell is the focal adhesion area where the cytoskeleton inside the cell connects to the extracellular environment through transmembrane proteins. To study mechanotrasduction, scientists normally prod the focal adhesion area with tiny protruding objects, such as pipettes or atomic force microscope tips. “This physical approach, typical of the mechanobiology community, limits the possibility to study mechanotrasduction in tissues.” says Aránzazu del Campo from INM-Leibniz Institute for New Materials in Germany. She and her collaborators therefore set out to explore the use of artificial molecular motors to induce a mechanical response in cells in vitro^1^. “Calculations had suggested that the force could be within the range for triggering a response, though it was unclear if the exposure dose needed would be compatible with living systems.” continues del Campo.

In one experiment, the researchers used photoactivated overcrowded alkene-based molecular motors embedded on a polymeric matrix^2^. Polymer appendices are attached to the focal adhesion area. Light irradiation makes the molecular motor rotate onto itself causing entanglement of the polymer chains, which in turn exert a pulling force to the transmembrane proteins (Fig. 1). The force is in the range of tens of piconewton and can be tuned by the light intensity or the duration of the irradiation. The position of the mechanical stimulus depends on the size of the irradiated area. Del Campo and collaborators measured the area of the focal adhesion region, and followed it grow overtime, as a result of the biochemical response induced by the mechanical stimulus.

**Fig. 1:**
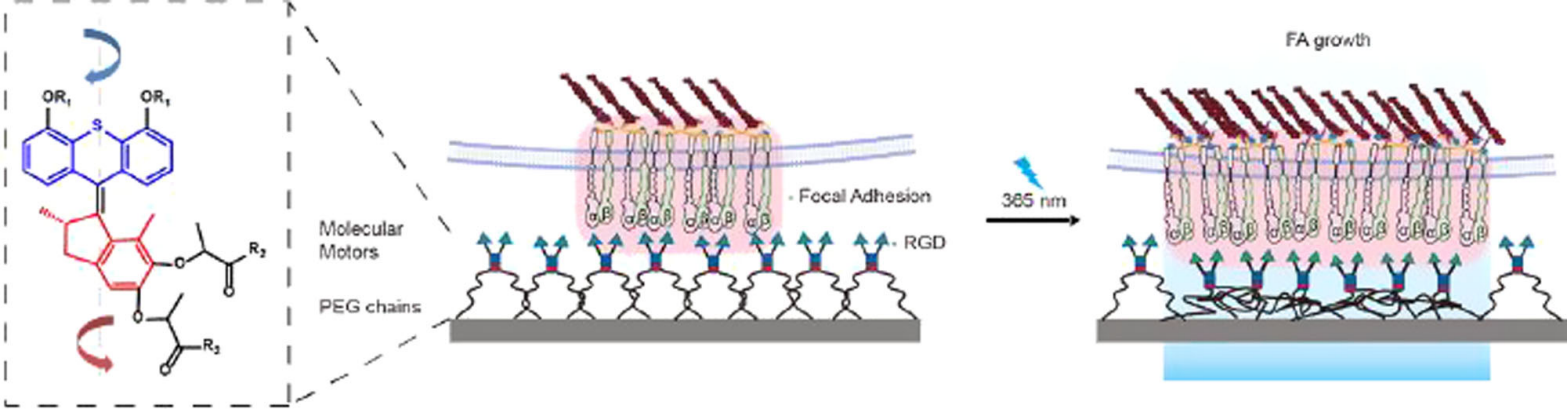
Mechanical force on focal adhesion area by molecular motors. Artificial molecular motors are conjugated to a polymeric, PEG matrix and bind to the focal adhesion region of a cell (left). Upon irradiation by UV light, the motor units (blue and red) turn with respect to one another inducing the polymer chains to entangle and shrink. As a result, the focal adhesion area increases (right). This areal increase is the result of the biochemical response of the cell to the mechanical stimulus by the motors^1^. Copyright: Modified from ref. ^1^, used under CC BY.

“These experiments are extremely delicate, because they require applying and measuring forces at the nanometer scale. In particular, we needed to demonstrate that the cellular response we observed was solely due to motor-driven forces.” explains del Campo.

The study is a proof-of-principle demonstration that photoactivated molecules, external to the cellular environment, can exert enough force on a cell surface to induce a biochemical response. Del Campo and colleagues now want to target more complicated systems, such as full tissues, where the study of mechanotrasduction at the nanometer scale by physical probing fails.
